# Sorption of Heavy Metals by Sewage Sludge and Its Mixtures with Soil from Wastewater Treatment Plants Operating in MBR and INR Technology

**DOI:** 10.3390/membranes11090706

**Published:** 2021-09-14

**Authors:** Robert Kowalik, Małgorzata Widłak, Agata Widłak

**Affiliations:** 1 Faculty of Environmental, Geomatic and Energy Engineering, Kielce University of Technology, al. Tysiąclecia Państwa Polskiego 7, 25-314 Kielce, Poland; rkowalik@tu.kielce.pl; 2 Hilti Entwicklungsgesellschaft mbH—Health, Safety and Environment, 86916 Kaufering, Germany; widlak.agata@gmail.com

**Keywords:** sewage sludge, sewage sludge-soil mixture, sequential extraction, heavy metals, sorption

## Abstract

Sewage sludge is a very complex system, with solids and water. It is generated as waste from wastewater treatment. Sewage sludge is used to fertilize agricultural and forest areas and to rehabilitate devastated areas. It is a good organic fertilizer because it contains significant amounts of nutrients beneficial for plant development and humus-forming substances. The composition of sludge from municipal wastewater treatment plants is similar to soil organic matter, therefore it can be used to improve the physicochemical properties of soil, increasing its sorption capacity. Research material was collected in the Swietokrzyskie and Mazowieckie Voivodships. Sewage sludge was collected from the wastewater treatment plants in Sitkowka Nowiny (Sitkowka) and Kunow, as well as high-quality agricultural soil from Opatowiec and sandy-clay soil from Jastrzebie. Research was carried out on the sorption of heavy metals (Cd, Cr, Cu, Pb, Ni, Zn) by mixtures of sewage sludge with soil. The calculations were made for the concentrations of heavy metals in sewage sludge, soil, and sewage sludge–soil mixtures. The geoaccumulation index (Igeo) and the risk assessment code (RAC) were calculated. Increased sorption capacity was demonstrated in samples with a predominance of sewage sludge. It was shown that heavy metals from sewage sludge, after mixing with soil, changed their form from immobile to mobile.

## 1. Introduction

The rapid pace of the development of civilization and industry causes the degradation of the natural environment, and special attention should be paid to large urban agglomerations, areas around industrial plants, and also a number of small towns. Society is still exposed to various pollutants that reach us from almost everywhere. Our health is exposed to the influence of polluted air, soil, water, and chemical substances contained in food [1,2].

Soil is a natural resource necessary for the survival of human life, but it accumulates toxic compounds (heavy metals), and there is a high probability of these pollutants getting into groundwater and the trophic chain. The consequence is the disturbance of the proper functioning of the ecosystem. The high demand for food requires intensive fertilization for better, faster, more efficient, and at the same time affordable, agricultural production. A favorable solution may be the use of sewage sludge in agriculture, because it is the cheapest method for their disposal and allows for a significant reduction in the consumption of mineral fertilizers. It is assumed that if the content of heavy metals in these sediments is lower than or equal to the permissible value, then they lose the features of the sewage sludge and can be used as mineral-organic fertilizer [3,4].

The choice of the method of sewage sludge management is dictated, in particular, by its quantity and properties [5,6,7]. In addition, this is subject to legal regulations. In Poland, the Act on waste [8], the ordinance of the Minister of the Environment on municipal sewage sludge [9], and the ordinance of the Minister of Economy on the criteria and procedures for allowing waste to be deposited at a given type of landfill are in force [10]. In Europe, heavy metal limits in terms of natural use are regulated by the Council Directive of 12 June 1986 on the protection of the environment, in particular soil, when sewage sludge is used in agriculture [11]; in the United States it is the Code of Federal Regulations [12]; for China, the 2002 Municipal Wastewater Discharge Standard [13]; and for South Africa, Guidelines for the use and disposal of sewage sludge [14]. The limits of heavy metals content in sewage sludge intended for natural use are presented in Table 1.

The presence of heavy metals in sewage sludge, and especially their high concentration, results from the share of industrial sewage (e.g., tannery, varnish, metallurgical sewage) in the total mass of municipal sewage. Moreover, heavy metals come from domestic sewage, surface runoff, and occur in sewage as a result of the corrosion of pipes [15].

To determine the potential risk of ecological soil contamination with heavy metals, two indicators were used. The geoaccumulation index (Igeo), which predicts the risk of contamination by comparing the content of heavy metals in the sewage sludge and the soil on which it will be applied [16,17]. The second indicator was the risk assessment code (RAC), which takes into account the mobility of heavy metals, using only the FI fraction, which migrates most towards the soil [18,19].

The total content of heavy metals in sewage sludge does not give information on the amount of metals available to plants. Thus, it is not possible to determine how much will enter the trophic chain. The use of sewage sludge often limits, or prevents, heavy metal content [20,21]. By using modern sequential analysis methods, we can estimate the percentage of metals in mobile form and determine the conditions for adsorption and desorption of metals in appropriate proportions of sludge–soil mixtures.

The concept of sorption covers two phenomena: absorption, which is the ability of a substance to be absorbed by the entire volume of another substance (the absorbent); and desorption, which results in compaction of the sorbed substance only on the surface of the adsorbent [22,23]. 

The sorption capacity of soils and the ground results from the fact that they are three-phase complexes, consisting of mutually interacting phases: solid, liquid, and gas. Sorption processes taking place in soils and grounds are mainly connected with the phenomenon of adsorption occurring at the border of two phases: solid and liquid. Physical adsorption is influenced by van der Waals forces and hydrogen bridge bonds [22,24]. Soils can retain whole particles of, for example, oxygen, carbon dioxide, or ammonia. The particles retained on the surface can reach energies that exceed the binding energies. This is called desorption. In chemical sorption substances are sorbed due to the formation of covalent bonds with some ionic bonding, and as a result the sorbent changes its properties and chemical composition [25]. 

The research team of scientists from the Kielce University of Technology was the first to test the content of heavy metals in sewage sludge from a wastewater treatment plant with the latest MBR technology. This new approach has significance for technological progress and sustainability. It has been shown that the high content of heavy metals in these sediments is mainly due to the fact that the MBR technology is BAT. This means that the membranes integrated in the process line retain much more heavy metals than their classic counterparts; therefore, their values in the sewage sludge are increased.

## 2. Materials and Methods

**Figure 1:**
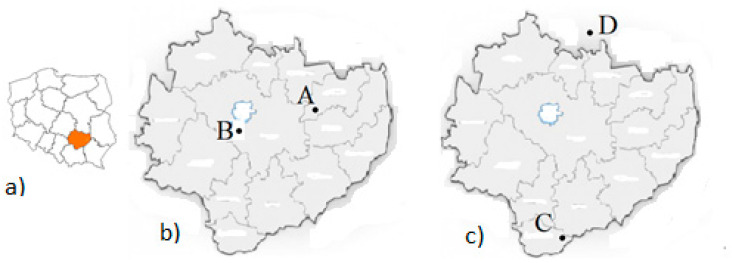
Location of sampling points: (**a**) Poland, Swietokrzyskie Voivodship; (**b**) location of the wastewater treatment plants in the Swietokrzyskie Voivodeship: A. Kunow, B. Sitkowka; (**c**) Jastrzab. Location of soil sampling in the Swietokrzyskie and Mazowieckie Voivodeships: C. Opatowiec, D. Jastrzab.

Municipal sewage sludge, collected in accordance with the guidelines on sampling sludges from sewage and water treatment works [26], was used for this research from two wastewater treatment plants in the Swietokrzyskie Voivodeship. The first, the Sitkowka Nowiny, is a mechanical-biological wastewater treatment plant operating based on INR technology (increased biogen removal) in the Nowiny commune (Figure 1). The river Bobrza is the receiver of the sewage.

The second wastewater treatment is the Kunow plant, in the Kunow commune, which works with MBR (membrane biological reaktor) technology, based on membrane ultrafiltration; ensuring highly effective treatment of sewage supplied to, and flowing through, the sewage system. Membrane wastewater treatment systems (MBR reactors) are used for very high requirements of treated wastewater. These are the most modern methods of treatment, the basis of which is the filtration of biologically treated wastewater through membranes (Figure 1b(B)). The Kamienna River is the receiver of the sewage.

The load expressed by the equivalent number of inhabitants for the Sitkowka wastewater treatment plant is 266,000, for the Kunow treatment plant it is 6687 [27,28]. Sewage sludge was used to prepare mixtures of sludge with soil. For this purpose, arable land was used. The mixtures were made in the proportions of sewage sludge to soil of 1:1, 1:2, and 2:1. The total mixtures of 60 g were left for 1 month in laboratory conditions. Air-dried samples were tested using the BCR sequential extraction method (Table 2) [29].

stage I: extraction CH_3_ COOH: for the determination of the content of assimilable and carbonate-bound metals (fraction FI: interchangeable, mobile),
stage II: extraction NH_2_OH·HCl: for the determination of the content of metals associated with amorphous iron and manganese oxides (fraction FII: reductive, mobile),
stage III: extraction H_2_O_2_/CH_3_COONH_4_: for the determination of the content of the organometallic and sulphide fraction (fraction FIII: oxidising, potentially mobile)
stage IV: mineralisation of the residual fraction with a mixture of concentrated acids (HCl, HF, HNO_3_): for the determination of the content of metals bound to silicates (fraction FIV: residual, immobile.

The content of heavy metals in the obtained extracts was determined in accordance with ISO 9001: 2000 on a Perkin-Elmer 3100 AAS-BG ICP-AES atomic absorption spectrophotometer (PerkinElmer, Waltham, MA, USA) after mineralization of the samples with aqua regia according to EN-13346: 2000] [29]. The reference sample consisted of 25 basic samples prepared from sludge–soil mixes.

For the obtained results, the arithmetic mean was calculated and the relationship between the content of heavy metals in the soils, sewage sludge, and sewage sludge–soil mixes from biological–mechanical and membrane treatment plants, as well as the geoaccumulation index (Igeo) and risk assessment code (RAC), were determined. The statistical analysis of the results was performed in Microsoft Excel 2010.

Geoaccumulation index of heavy metal in soil (Igeo)

The Igeo is used in order to assess the degree of accumulation of heavy metals. Originally, the I_GAI_ was used for the ecological risk assessment of bottom sediments [32]. It is also used for the assessment of the contamination of soil [16], sewage sludge, and sewage sludge ash [32]. The Igeo is described in the equation [17,19]:

(1) $$\mathrm{Igeo}\;=\log_{2}\frac{C_{n}}{1.5\cdot B_{n}}$$

where 

*C_n_* = content of a given element from the group of heavy metals contained in sewage sludge mg/kg d.m. 

*B_n_* = content of a given element from the group of heavy metals present in the soil, mg/kg d.m. 

The classification of heavy metal Igeo is: <0 = no pollution; 0–1 = no pollution, moderate pollution; 1–2 = moderate pollution; 2–3 = moderate or high pollution; 3–4 = high pollution; 4–5 = high or very high pollution; >5 = very high pollution [17].

Risk assessment code (*RAC*)

The *RAC* is also used to assess the environmental risks posed by heavy metals. The *RAC* has been used to assess soil contamination with heavy metals from sewage sludge and sewage sludge ashes [19]. The *RAC* takes into account the percentage of heavy metals present in the mobile fraction (*F*1). The risk level can be classified into 5 categories: <1 = no risk; 1–10 = low risk; 11–30 = medium risk; 30–50 = high risk; >50 = very high risk [18]. It is defined by the formula [16,17]:

(2) $$RAC=\frac{F1}{HM}\cdot 100\%$$

where

*F*1 = concentration of heavy metal in acid–soluble/free fraction; *F*1, mg/kg; *HM* = total heavy metal concentration, mg/kg.

## 3. Research Results and Discussion

### 3.1. Soil

Samples for laboratory chemical analysis were taken from the soil with an Egner cane at a depth of up to 30 cm [20]. Soil from Opatowiec in the Kazimierz Wielka poviat, in the Swietokrzyskie Voivodship, was used for the tests. It is a typical agricultural area, with soils of a very good wheat complex, and with 75% classified as chernozem class I and II: brown soils, humus-rich bogs. These soils are the most rich in nutrients (Figure 1) [33].

Heavy metals in the soil from Opatowiec, both for individual fractions and the total composition, did not exceed the applicable standard in relation to all metals, except for cadmium Cd (Table 3). In the mobile fractions: FI-acetate, cadmium constituted 64% of the norm; in FII-carbonate it was 16% and in FIV-organic 65% of the norm. The exceding of the applicable standard for cadmium occurred in the FIII-oxide fraction, 37% (Table 3) [34].

**Figure 2:**
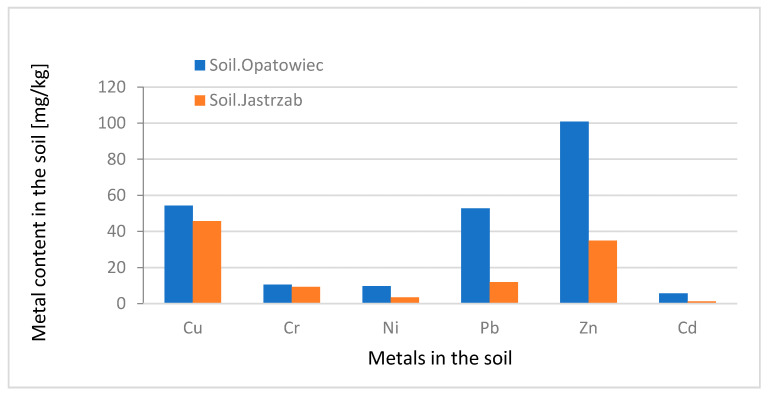
Total metal content in the soil from Opatowiec and Jastrzab (mg/kg d.m.).

The soils of the Jastrzab commune are of a low quality for agricultural production; the share in individual valuation classes is 74% for classes V and VI. These classes include rocky or sandy soils with a low level of humus. They are poor in organic substances (Figure 1). The fractions FI–FIV showed the content of heavy metals was up to 30% over the applicable standard; for mobile fraction FI-acetate 8.5%, and FII-carbonate 17.7% (Table 4) [35]. In the mobile fractions FI and FII in the soil from Jastrzab, the % content of metals Cu, Cd, Ni, Pb, and Zn was in the range of 43–59%, in the soil from Opatowiec the % content in this range was Ni and Zn (Table 5, Figure 2).

### 3.2. Sewage Sludge

The study used samples of sewage sludge collected from two different wastewater treatment plants located in the Swietokrzyskie Voivodeship (Figure 1). The first facility, the wastewater treatment plant in Kunow, uses the technology of biological membrane reactors. MBR technology is recognized as the best possible technology for wastewater treatment (BAT = best available technology) within the meaning of the IPPC Directive, Integrated Pollution Prevention and Control [36,37]. Ultrafiltration on membranes allows maintaining in the biological reactor a many times higher concentration of activated sludge (8–12 g/m^3^) than is allowed for secondary settling tanks. With MBR technology there is no need to use preliminary settling tanks. High concentrations and great age of the sludge do not threaten its overload. Such conditions also give a more efficient nitrification process, as well as low sludge growth, which results in smaller amounts of excess sludge requiring processing and management [38,39]. The wastewater treatment plant in Kunow is equipped with a solar sewage sludge dryer, which uses only solar energy in the drying process. The collected sludge was dried and dehydrated.

The second facility was the Sitkowka wastewater treatment plant, operating with three-phase activated sludge technology with increased removal of biogenic elements. The sludge is anaerobically stabilized by methane fermentation (WKFz), thickened in a sludge thickener, and then dewatered in centrifuges. Since 2012, the sludge has been neutralized at the Thermal Sewage Sludge Treatment Station located in the Sitkowka treatment plant. The sludge sample for testing was collected after the dewatering processes in centrifuges. Afterwards it was dried in a Labotherm Nebertherm laboratory oven; the loss on ignition (LOI) of the sludge was 63.1%.

**Figure 3:**
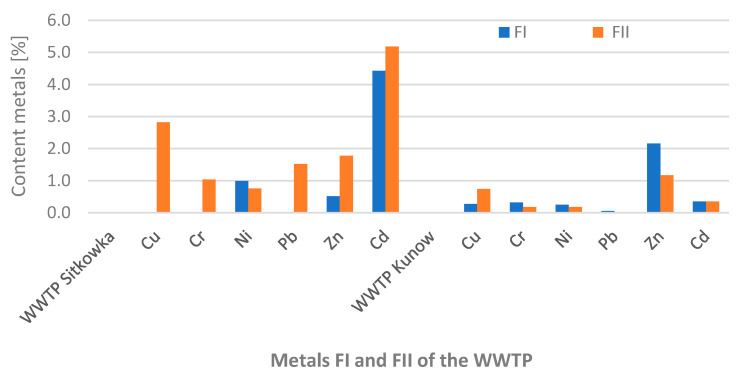
Percentage % of metals in the FI and FII fractions of the sludge of WWTP Sitkowka and WWTP Kunow in relation to the Polish standard [9].

**Figure 4:**
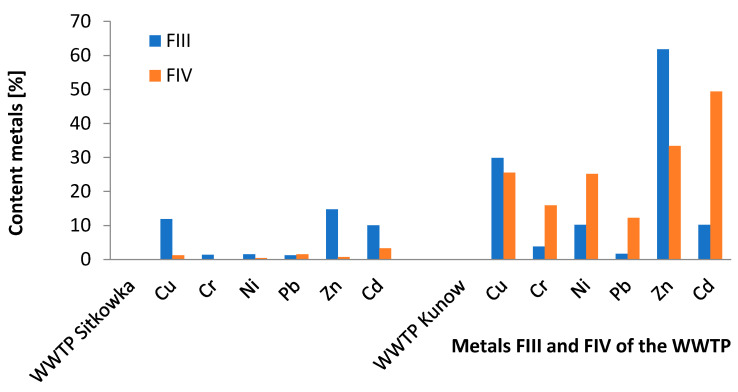
Percentage of metals in the FIII and FIV fractions of the sludge of WWTP.

The speciation analysis of sewage sludge collected from the wastewater treatment plant in Sitkowka showed that the standards for heavy metal content were not exceeded (Table 6 and Table 7). The highest concentrations of copper, nickel, chromium, cadmium, and zinc were recorded for Fraction III; respectively, Cu 74.46%, Ni 40.89%, Cr 57.55%, Cd 43.67%, and Zn 79.86%. On the other hand, lead dominated in fraction IV (35.81%) in relation to the total content (Table 6). In the tested sewage sludge in fractions FI and FII (mobile fractions) from the Sitkowka wastewater treatment plant, copper, zinc, and lead were dominant in the range not exceeding 5% in relation to the standard in force in Poland (Figure 3) [9]. In the case of FIII (oxide fraction), the copper, zinc, and cadmium metals were at up to 15% of the applicable standard (Table 7, Figure 4) [9].

Sewage sludge from Kunow did not exceed the limits of heavy metals content; only zinc was on the border of the limit value of the applicable standard [9] and amounted to 2463.46 mg/kg d.m., of which 62.71% was contained in fraction FIII (Table 7 and Table 8). For chromium, cadmium, nickel, and lead, the highest content was recorded for fraction FIV: respectively, Cr 80.64%, Cd 82.40%, Ni 70.31%, and Pb 87.6%. On the other hand, copper dominated in fraction FIII (52.92%).

The Kunow wastewater treatment plant with membrane wastewater treatment systems ensures the effective treatment of heavy metals in the mobile fraction FI of up to 98%, and of FII up to 99% (Table 8, Figure 3). The FIII-oxide fraction contained 62% zinc and the FIV-organic fraction 49% cadmium (Table 8, Figure 4) [9].

The aim of the study was to assess the sorption capacity of sewage sludge and sludge–soil mixtures in relation to heavy metals from two wastewater treatment plants (Sitkowka and Kunow) and two types of agricultural soil from Opatowiec (very good) and Jastrzebie (medium). Mixtures of sludge from Kunow with soil from Opatowiec and of sludge from Sitkowka with soil from Jastrzebie were used for the study. The mixtures were created in sediment:soil ratios of 1:1, 1:2, and 2:1.

**Figure 5:**
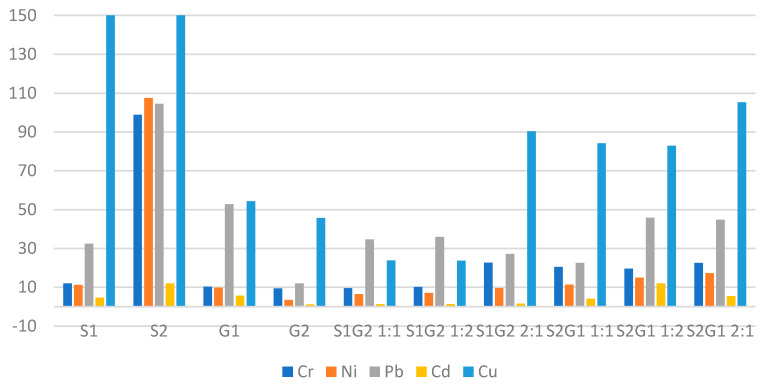
Heavy metal content of sludge, soils, and sludge–soil mixtures (mg/kg d.m.) (S1 Sitkowka, S2 Kunow, G1 Opatowiec, G2 Jastrzab).

**Figure 6:**
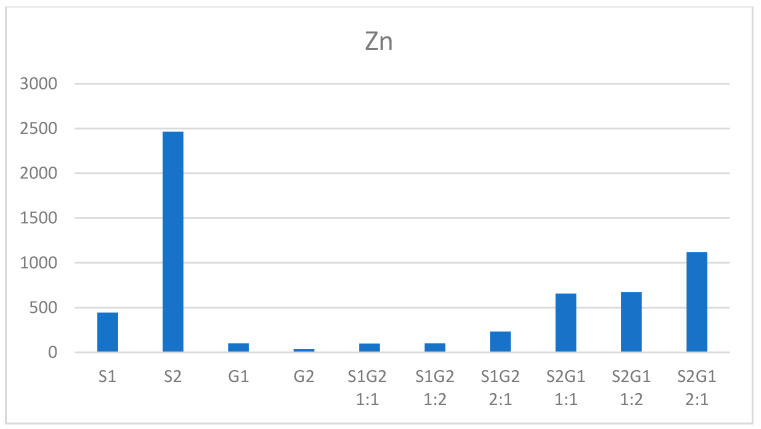
Heavy metal content of sludge, soils, and sludge–soil mixtures (mg/kg d.m.) (S1 Sitkowka, S2 Kunow, G1 Opatowiec, G2 Jastrzab).

The soil–sediment mixtures in 1:1 and 1:2 ratio showed comparable sorption properties (Figure 5). The highest sorption capacity was found in the samples of mixtures 2:1 (Figure 5 and Figure 6).

The metals Cr, Ni, Pb, and Cd found in the studied mixtures in sediment and soil ranged from 0.542 to 107.54 mg/kg d.m., while Cu ranged from 23.81 to 564.37 mg/kg d.m. (Figure 5). Zinc, on the other hand, significantly exceeded the results obtained for the other metals, ranging from 34.84 mg/kg d.m. to 2463.47 mg/kg d.m. (Figure 6).

**Figure 7:**
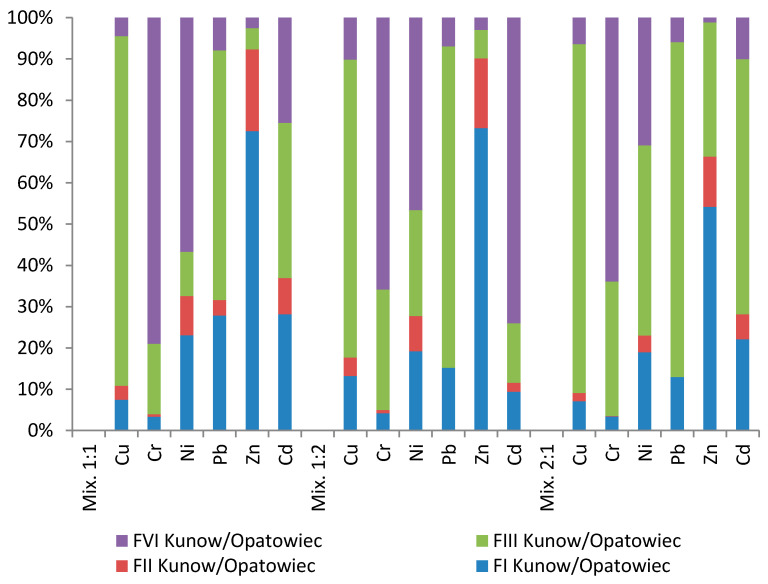
Percentage contents of heavy metals for particular fractions in Kunow–Opatowiec sludge–soil mixtures.

The speciation analysis of heavy metals in the mixtures showed a tendency to change the form of heavy metal occurrence, from immobile to mobile. This was particularly observed in the case of zinc in Kunow–Opatowiec mixtures. Zinc content in the FI fraction, the most mobile, oscillated within 55% for the 2:1 mixture and 72–74% for the 1:1 and 1:2 mixtures (Table 8, Figure 7). In contrast, the percentage of zinc FI for the sediment was less than 0.02%. The remaining heavy metals in case of the Kunow–Opatowiec mixtures remained mostly in the immobile fractions FIII and FIV (Table 9, Figure 7).

**Figure 8:**
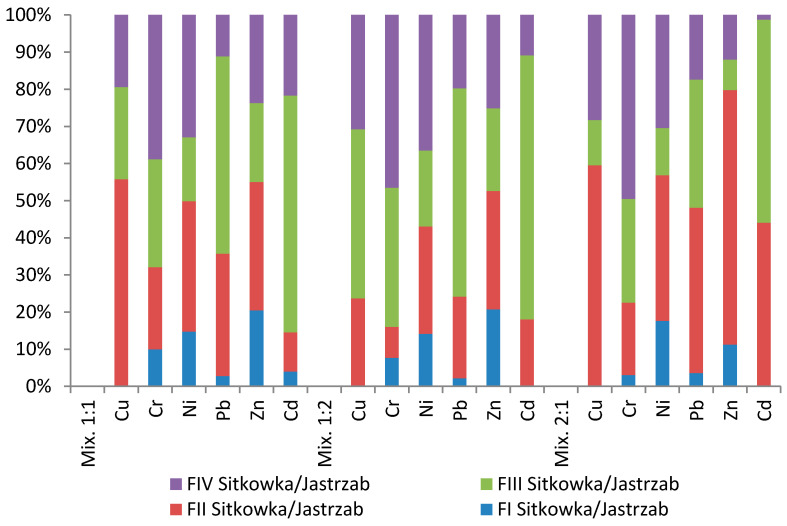
Percentage contents of heavy metals for particular fractions in Sitkowka–Jastrzab sludge–soil mixtures.

Heavy metals in the Sitkowka–Jastrzab mixtures dominated fractions FII and FIII. The highest percentage for the mobile fraction FII was recorded for copper; it was respectively 55–60% for 1:1 and 2:1 mixtures, and 23.83% for 1:2 FIV (Table 9, Figure 8). Zinc dominated the FII fraction, being 34.57% for 1:1, 31.9% for 1:2, and 68.58% for 2:1, respectively.

Indicators for environmental contamination risk analysis.

**Figure 9:**
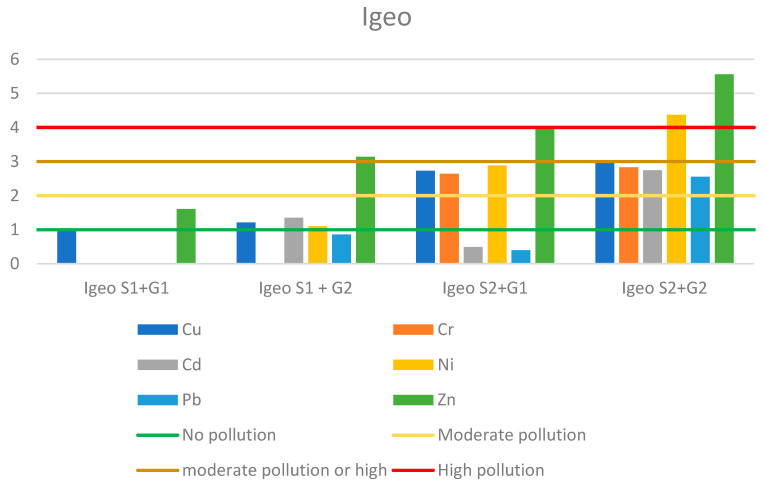
Value of geoaccumulation index (Igeo) in sludge–soil mixtures. (S1 Sitkowka, S2 Kunow, G1 Opatowiec, G2 Jastrzab).

A simulation analysis of heavy metal contamination of soil after application of sewage sludge was performed using the Igeo index. Calculations were made for four variants: sludge from the Sitkowka treatment plant (S1) on soil from Opatowiec (G1) and Jastrzab (G2), and sludge from the Kunow treatment plant (S2) on soil from Opatowiec (G1) and Jastrzab (G2). The lowest contamination risk was found when sludge S1 was applied on G1; only zinc showed a moderate risk, while the other metals showed no contamination (Figure 9). The highest potential risk of environmental contamination was found for S2 on G2. Zinc and nickel indicated a very high risk of contamination; the other metals were at high risk (Figure 9). Zinc was dominant in all cases. Analyzing the results of the Igeo index, it can be concluded that the use of sewage sludge from the MBR treatment plant carries a higher risk of environmental contamination than sludge from IBR technology.

**Figure 10:**
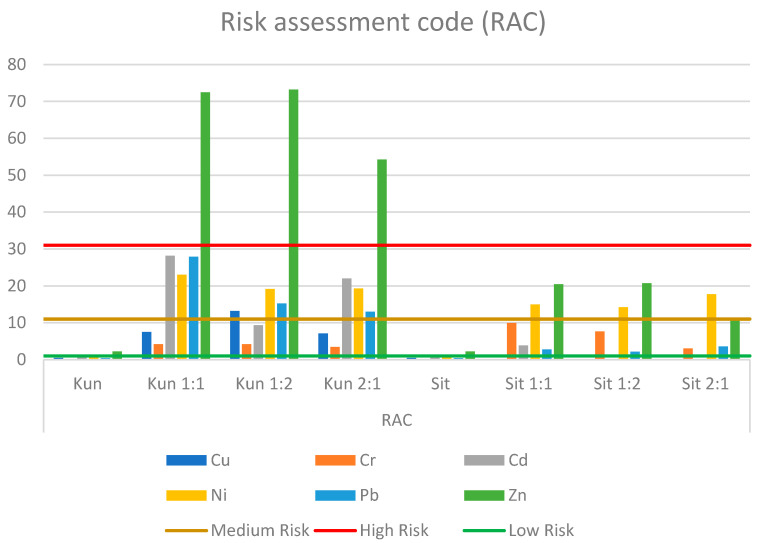
Value of RAC index in sludge–soil mixtures.

In most cases, the level of the RAC indicator does not show a high environmental risk for the sludge from both WWTPs (Figure 10). However, when analyzing the risk assessment code for the sludge–soil mixtures, it can be seen that the value of the indicator mostly shows a medium or high risk of contamination. This is due to the high metal content of the mixtures in the FI fraction, the most mobile. The highest exceedances were recorded for zinc from Kunow–Opatowiec mixtures, it was respectively 72.5% for the 1:1 mixture, 73.23% for 1:2, and 54.22% for 2:1 (Figure 10). Cadmium, lead, and nickel for the 1:1 and 2:1 mixtures, and copper, nickel, and lead for the 1:2 mixture also posed an average risk of contamination. The Sitkowka–Jastrzab mixtures showed definitively lower risk of contamination. Zinc was also found to be the dominant metal in most cases; being 20.44% for 1:1, 20.73% for 1:2, and 11.24 for 2:1, respectively. In all three mixtures, nickel also showed an average risk and was found to be 14.93% for 1:1, 14.21% for 1:2, and 17.72% for 2:1, respectively (Figure 10).

## 4. Conclusions

The research material was collected in the Swietokrzyskie and Mazowieckie Voivodships. The sewage sludge was collected from the wastewater treatment plants in Sitkowka and Kunow, as well as high-quality agricultural soil from Opatowiec and sandy-clay soil from Jastrzab. Soil from Opatowiec and soil from Jastrzab had a comparable content of copper (Cu), at the level of 25% in relation to the applicable standard in Poland.

Soil from Opatowiec has a higher zinc (Zn) content than soil from Jastrzab due to differences in soil quality classes. In the sludge of the Sitkowka WWTP and in the sludge of the Kunow WWTP, heavy metals (Cd, Cr, Cu, Pb, Ni, Zn) predominate in the immobile FIII and FIV fractions. In the FI and FII fractions in the sludge of the Sitkowka WWTP, and in the sludge of the Kunow WWTP, the highest content was found for zinc, but at the level of 1.2–2.2% of the applicable standard. The samples in which sediment predominated showed the highest sorption capacity. Mixtures in a 1:1 and 1:2 ratio showed comparable sorption properties. Heavy metals in the Kunow–Opatowiec mixtures tended to change their form, from immobile to mobile.

In the Sitkowka–Jastrzab mixtures, heavy metals dominated in fractions FII and FIII. The highest percentage content for the mobile fraction FII was recorded for copper.

The analysis of the risk of heavy metal contamination of the environment using the Igeo index showed a significantly higher risk when using sludge from the wastewater treatment plant operating with MBR technology. The high heavy metal content of this sludge is mainly due to the fact that the MBR technology is BAT. This means that the membranes integrated in the process line retain considerably more heavy metals than their classical counterparts, and therefore their values in the sewage sludge are increased. 

The RAC risk assessment code also showed a higher risk for the Kunow–Opatowiec sludge–soil mixture than for the Sitkowka–Jastrzab, especially for zinc, due to the fact that there was a change in the form of heavy metals from immobile to mobile.

Sewage sludge from the Kunow wastewater treatment plant operating with MBR technology and the Sitkowka wastewater treatment plant operating with INR technology can be used for agricultural purposes, with the application of an appropriate amount of sewage sludge, corresponding to a 1:1 mixture of sewage sludge and soil. The application of sludge requires close cooperation with farmers using agricultural fertilization supported with sludge from wastewater treatment plants.

## Figures and Tables

**Table 1:** Normative limit values of heavy metals in sewage sludge for natural use (mg/kg d.m.).

Metal	Permissible Values for Heavy Metals Intended for Natural Use					
Region	Poland Regulation [9]	EU Directive 86/278/EEC [11]	Chinese Regulation GB 18918-2002 [13]		USA Regulation 40 CFR Part 503, 503.13 [12]	South African Guideline (Pollutant Class a) [14]
			pH < 6.5	pH > 6.5		
Cd	20	20–40	5	20	39	40
Ni	300	300–400	100	200	420	420
Zn	2500	2500–4000	500	1000	2800	2800
Cu	1000	1000–1750	250	500	1500	1500
Cr	500	-	600	1000	-	1200
Pb	750	750–1200	300	1000	300	300

**Table 2:** Method of metal speciation of heavy metals in sewage sludge and soil [30,31].

Fraction	Form of Metal	Parameters of Fractionation	Time of Extraction, h
FI	Carbonate bound	0.11 M CH_3_COOH, pH = 7.0, T = 20 °C	16
FII	Fe/Mn oxides bound	0.1 M NH_2_OH∙HCl pH = 2.0	16
FIII	Organic	30% H_2_O_2_ + 8.8 M H_2_O_2_ pH = 2.0, T = 85 °C	16
FIV	Residual	10 M HNO_3_ + 10 M HCl, T = 100 °C	3

**Table 3:** Chemical speciation of heavy metals in the soil from Opatowiec in the Swietokrzyskie Voivodeship (mg/kg).

Opatowiec Soil						
Fraction	Cu	Cr	Cd	Ni	Pb	Zn
Fraction I	4.04	0.43	1.27	3.45	8.32	48.55
Fraction II	1.22	0.08	0.32	0.68	5.76	11.76
Fraction III	34.24	3.00	2.74	1.19	32.30	13.58
Fraction IV	14.75	7.00	1.32	4.37	6.34	26.89
Σ FI-FIV	54.26	10.52	5.67	9.71	52.73	100.79
Permissible contents of selected metals in soil [10]	200	200	2	150	200	500

**Table 4:** Chemical speciation of heavy metals in the soil from Jastrzab in Mazowieckie Voivodship (mg/kg).

Jastrzab Soil						
Fraction	Cu	Cr	Cd	Ni	Pb	Zn
Fraction I	0.23	0.01	0.17	0.44	0.37	10.64
Fraction II	25.70	0.51	0.35	1.05	6.03	9.64
Fraction III	17.07	0.72	0.54	0.51	5.23	6.98
Fraction IV	2.73	8.15	0.13	1.45	0.25	7.59
Σ FI-FIV	45.74	9.25	1.20	3.47	11.89	34.87
Permissible contents of selected metals in soil [10]	200	200	2	150	200	500

**Table 5:** Percentage % of heavy metals of the FI and FII fraction in the Jastrzab and the Opatowiec soils in relation to the total content of ∑FI-FIV metals.

	Jastrzab Soil					
Metals	Cu	Cr	Cd	Ni	Pb	Zn
Fraction I (mg/kg)	0.23	0.01	0.17	0.44	0.37	10.64
Fraction II (mg/kg)	25.70	0.51	0.35	1.05	6.03	9.64
∑FI-FIV (mg/kg)	45.74	9.25	1.20	3.47	11.89	34.87
Content % ∑FI-FII	56.7	5.5	43.6	43.1	53.9	58.2
	**Opatowiec Soil**					
Fraction I (mg/kg)	4.04	0.43	1.27	3.45	8.32	48.55
Fraction II (mg/kg)	1.22	0.08	0.32	0.68	5.76	11.76
∑FI-FIV (mg/kg)	54.26	10.52	5.67	9.71	52.73	100.79
Content % ∑FI-FII	9.7	5.1	28.2	42.7	26.7	59.8

**Table 6:** Chemical speciation of heavy metals in sewage sludge from the Sitkowka wastewater treatment plant in the Swietokrzyskie Voivodship (mg/kg).

Sewage Sludge Sitkowka						
Fraction	Cu	Cr	Cd	Ni	Pb	Zn
Fraction I	0.0005	0.0001	0.8846	2.9742	0.1660	12.8170
Fraction II	28.1660	5.1707	1.0356	2.2629	11.376	44.472
Fraction III	118.5600	6.7822	2.0024	4.5863	9.2669	368.74
Fraction IV	12.504	0.0001	0.6629	1.3780	11.6150	17.694
Σ FI–FIV	159.2305	11.7859	4.5855	11.2014	32.4239	461.7230
Permissible contents of selected metals in sewage sludge PL [9]	1000	500	20	300	750	2500
Permissible content of selected metals in sewage sludge EU Directive 86/278/ EEC [11]	1000–1750	-	20–40	300–400	750–1200	2500–4000

**Table 7:** Percentage % of metals in the sludge in the FIII and FIV fractions in relation to the total content of Σ FI–FIV.

	Sewage Sludge Sitkowka					
Metals	Cu	Cr	Cd	Ni	Pb	Zn
Fraction III (mg/kg)	118.5600	6.7822	2.0024	4.5863	-	368.74
Fraction IV (mg/kg)	-	-	-	-	11.6150	-
∑FI-FIV (mg/kg)	159.2305	11.7859	4.5855	11.2014	32.4239	461.7230
Content % Σ FI–FIV	74.46	57.55	43.67	40.89	35.81	79.86
	**Sewage Sludge Kunow**					
Fraction III (mg/kg)	298.64	-	-	-	-	1544.97
Fraction IV (mg/kg)	-	79.71	9.88	75.60	91.60	-
∑FI-FIV (mg/kg)	564.36	98.84	11.99	107.53	104.57	2463.46
Content % Σ FI–FIV	87.60	80.64	82.40	73.31	87.6	62.71

**Table 8:** Chemical speciation of heavy metals in sewage sludge from the Kunow wastewater treatment plant in the Swietokrzyskie Voivodship (mg/kg).

Sewage Sludge Kunow						
Fraction	Cu	Cr	Cd	Ni	Pb	Zn
Fraction I	2.72	0.16	0.07	0.76	0.4	53.93
Fraction II	7.39	0.09	0.00	0.54	0.00	29.13
Fraction III	298.64	18.89	2.04	30.64	12.57	1544.97
Fraction IV	255.62	79.71	9.88	75.60	91.60	835.44
Σ FI–FIV	564.36	98.84	11.99	107.53	104.57	2463.46
Poland Regulation [9]	1000	500	20	300	750	2500
EU Directive 86/278/EEC [11]	1000–1750	-	20–40	300–400	750–1200	2500–4000

**Table 9:** Kunow–Opatowiec and Sitkowka–Jastrzab sludge–soil mixtures.

Kunow–Opatowiec Sludge–Soil Mixture						
Sludge–Soil Mixture 1:1 (mg/kg)						
Fraction	Cu	Cr	Cd	Ni	Pb	Zn
Fraction I	6.3006	0.6882	1.1430	2.6136	6.2827	474.2400
Fraction II	2.8508	0.1183	0.3559	1.0737	0.845	129.5600
Fraction III	71.3150	3.4836	1.5245	1.2174	13.609	33.6010
Fraction IV	3.7602	16.1250	1.032	6.418	1.7885	16.6800
Σ FI–FIV	84.2266	20.4251	4.0554	11.3227	22.5252	654.0810
**Sludge–Soil Mixture 1:2**						
**Fraction**	**Cu**	**Cr**	**Cd**	**Ni**	**Pb**	**Zn**
Fraction I	10.9610	0.8201	1.1266	2.8680	6.9879	491.6000
Fraction II	3.7213	0.1567	0.2648	1.2756	−0.0756	113.5600
Fraction III	59.8100	5.7016	1.72	3.8370	35.7680	46.1810
Fraction IV	8.4350	12.8660	8.8671	6.9639	3.1982	19.9250
Σ FI–FIV	82.9183	19.5444	11.9785	14.9445	45.8785	671.2660
**Sludge–Soil Mixture** **2:1**						
**Fraction**	**Cu**	**Cr**	**Cd**	**Ni**	**Pb**	**Zn**
Fraction I	7.4640	0.7759	1.1949	3.3227	5.8096	607.14
Fraction II	2.0925	0.0219	0.3276	0.7124	0.1338	135.86
Fraction III	89.0090	7.3406	3.3408	8.0772	36.2640	364.25
Fraction IV	6.7111	14.406	0.5420	5.4206	2.6401	12.525
Σ FI–FIV	105.2766	22.5444	5.4053	17.2359	44.7485	1119.775
**Sitkowka–Jastrzab Sludge–Soil Mixture**						
**Sludge–Soil Mixture 1:1**						
**Fraction**	**Cu**	**Cr**	**Cd**	**Ni**	**Pb**	**Zn**
Fraction I	0.00	0.9467	0.0519	0.9489	0.9652	20.077
Fraction II	13.332	2.0932	0.1382	2.2572	11.405	33.963
Fraction III	5.9293	2.7555	0.8308	1.1106	18.412	20.907
Fraction IV	4.6331	3.6822	0.2831	2.1165	3.86	23.283
Σ FI–FIV	23.8086	9.4776	1.304	6.4332	34.6422	98.23
**Sludge–Soil Mixture 1:2**						
**Fraction**	**Cu**	**Cr**	**Cd**	**Ni**	**Pb**	**Zn**
Fraction I	0.00	0.7745	0.00	1.0081	0.7691	20.8
Fraction II	5.6494	0.8435	0.242	2.0548	7.9252	32.012
Fraction III	10.875	3.7965	0.9522	1.4592	20.147	22.304
Fraction IV	7.3335	4.7013	0.1454	2.5945	7.0931	25.205
Σ FI-FIV	23.7151	10.1158	1.3248	7.1166	35.9344	100.321
**Sludge–Soil Mixture 2:1**						
**Fraction**	**Cu**	**Cr**	**Cd**	**Ni**	**Pb**	**Zn**
Fraction I	0.00	0.689	0.00	1.6698	0.9715	26.058
Fraction II	54.144	4.4099	0.7335	3.7157	12.119	158.98
Fraction III	11.079	6.327	0.9088	1.2052	9.3805	18.946
Fraction IV	25.681	11.214	0.0209	2.8823	4.7305	27.832
Σ FI–FIV	90.3562	22.6399	1.5214	9.473	27.2015	231.816

## Data Availability

The datasets supporting the results of this article are included within the article and its additional files.
